# An Intersectional Perspective on the Utilization of Cervical Cancer Screening among Migrants. A Cross-Sectional Analysis of Survey Data from Austria

**DOI:** 10.3390/cancers13236082

**Published:** 2021-12-02

**Authors:** Patrick Brzoska, Diana Wahidie, Yüce Yilmaz-Aslan

**Affiliations:** 1 Health Services Research, Faculty of Health, School of Medicine, Witten/Herdecke University, 58455 Witten, Germany; Diana.Wahidie@uni-wh.de (D.W.); Yuece.Yilmaz-Aslan@uni-wh.de (Y.Y.-A.); 2 Department of Epidemiology and International Public Health, School of Public Health, Bielefeld University, 33615 Bielefeld, Germany; 3 Department of Nursing and Health Services Research, School of Public Health, Bielefeld University, 33615 Bielefeld, Germany

**Keywords:** cervical cancer, screening, utilization, heterogeneity, migrants, Austria, survey

## Abstract

**Simple Summary:**

Studies from several countries have shown that migrant women utilize cervical cancer screening less frequently than non-migrant women. Little is known about how disparities differ across different countries of origin. The present study addresses this limitation by means of 2019 survey data from Austria. Comparing the five largest groups of migrants residing in the country, the results show that particularly Turkish migrant women have a lower utilization than the Austrian majority population. This illustrates the heterogeneity of migrants and likely results from different obstacles some groups of migrants encounter in the health system. The findings may contribute to raising the awareness of the heterogeneity of the migrant population and to providing cancer screening interventions tailored to different cultural backgrounds, consequently improving overall access to cancer screening for particularly disadvantaged and vulnerable population groups.

**Abstract:**

In most European countries, migrant women have lower rates of cervical cancer screening utilization than non-migrant women. While studies have illustrated that disparities can be partially explained by social determinants, they usually did not take into account the heterogeneity of the migrant population in terms of cultural background or country of origin. Applying an intersectional approach and using 2019 data from a representative survey from Austria on 6228 women aged 20–69 years, the present study examines differences in the utilization of cervical cancer screening in the five largest migrant groups (i.e., individuals with a nationality from or born in a Yugoslav successor state, Turkey, Romania, Hungary, or Germany) residing in Austria. By means of a multivariable analysis, amongst others adjusted for socioeconomic and health-related determinants, it is illustrated that particularly Turkish migrant women have a lower utilization than the Austrian majority population (adjusted odds ratio (OR) = 0.60; 95% confidential interval (CI): 0.40–0.91), while no significant differences between the majority population and other groups of migrants became evident. The findings are indicative of the heterogeneity of migrants and likely result from different obstacles some groups of migrants encounter in the health system. This heterogeneity must be taken into account in order to support informed decision-making and to ensure adequate preventive care.

## 1. Introduction

Cervical cancer accounts for about four percent of all cancer cases in Europe among women aged 20 to 69 years and is the seventh most common type of cancer among women in that age group [1]. Regular Papanicolaou (Pap) smear testing provides an effective method for the early detection of pre-cancerous lesions and is able to reduce the incidence and mortality of cervical cancer considerably [2]. With current guidelines slightly differing between countries, regular Pap smears are usually recommended for women starting with the age group of 20 or 30 years up to the age group of 60 to 69 years [3,4], with recommended screening intervals ranging between 3 and 5 years [5].

Across Europe, on average, only about 10.3% to 67.4% of women participate in cancer screening according to the respective guidelines in their countries of residence [6]. Similar to the utilization of other preventive services, the non-utilization of cervical cancer screening is associated with low socioeconomic status [7,8,9], limited health literacy [10,11], and higher age [12]. In addition, several studies conducted in Europe [13,14,15,16,17] have found lower cervical cancer screening attendance among migrant women as compared to that of the native majority populations. For instance, a register-based study from Sweden reports that only 49% of immigrant women born outside Sweden participated in cervical cancer screening between 1993 and 2005 as compared to 62% of Swedish women [13]. Similarly, survey data from the 2010–2012 Finnish Migrant Health and Well-being study and the 2011 National Health Survey show that 93.6% of native Finnish women had taken a Pap test in the past five years before the survey. The proportions were considerably lower among women of other origin, ranging from 78.6% among Russian to 40.8% among Somali women [15]. Comparable findings were reported from other European countries such as Denmark [17], Italy [14], and Norway [16], as well as from other regions of the world such as Canada [18,19], the United States [20], and Australia [21].

In Austria, about 24 percent of the population are migrants, i.e., individuals who do not have Austrian citizenship or who themselves or whose parents were born abroad [22]. Austrian social insurance provides free Pap smears to all women, including migrant women with a residence status. The utilization of cervical cancer screening every three years is recommended for women in the age group of 19 to 69 years [23]. In addition, starting in 2014, Austria has been the first country in Europe to offer a vaccination program against human papillomavirus (HPV) [24], which along screening can significantly reduce the incidence of cervical cancer on the population level [25,26]. Austrian social insurance offers HPV vaccination free of charge to girls and boys aged 9 to 12 years [27].

A study from Austria shows that migrant women from European Union (EU) and non-EU countries are less likely to undergo a Pap smear test compared to native-born Austrian women [28]. Similar to other studies in the field, however, this study was not able to take the diversity of the migrant population into account, as it only compared EU/non-EU migrants with non-migrants. Given the heterogeneity of the migrant population in terms of cultural background or country of origin, this is a significant limitation. Studies from other countries not only show that the incidence of cervical cancer varies between different groups of migrants [29], but also reveal that this population is heterogeneous in terms of their preventive behavior and that also disparities as compared to non-migrants may differ. For example, studies on tertiary prevention in Germany reveal a lower utilization for Turkish migrants and migrants from EU countries, whereas German resettlers showed a higher utilization of tertiary prevention than non-migrants. The differences between EU and Turkish migrants compared to non-migrants also varied with age. While for Turkish migrants they decreased with age, they increased with age for EU migrants. No significant interaction effects were observable for other population groups [30]. This indicates the need for analyses across individual groups of migrants, which also take into account intersectional differences.

Using the data from a population-based survey and applying an intersectional approach, the aim of the present study was to investigate differences in the utilization of regular Pap smears in the five largest migrant groups residing in Austria: individuals with a nationality from a Yugoslav successor state, Turkey, Romania, Hungary, and Germany [22]. Insights gained by the study may also contribute to achieving one of the 90–70–90 goals of the WHO global strategy, i.e., to have 70% of women aged 35 to 45 years participate in cervical cancer screening by 2030 [31].

## 2. Materials and Methods

### 2.1. Data

The study uses data from the Austrian Health Interview Survey 2019, a cross-sectional survey conducted by Statistics Austria on behalf of the Federal Ministry of Social Affairs, Health, Care and Consumer Protection between October 2018 and September 2019. Based on the European Health Information Survey program, data collection was conducted through computer-assisted personal interviewing and computer-assisted web interviewing in combination with a questionnaire completed by respondents themselves. The sample comprised a total of 15,461 randomly selected individuals aged 15 years or older. Participation in the survey was voluntary, and all participants provided informed consent before participation. The response rate was 50.5% [32]. Aside from information on income, for which 8.8% of respondents did not provide information, the proportion of missing values was low. All missing values were imputed by Statistics Austria prior to the publication of the dataset. Imputation was based on the k-nearest neighbors algorithm [33] using sex, age, the level of education, the regional context, and other information as distance variables [34].

Corresponding to the age groups for which cervical cancer screening is recommended in Austria, we included women aged 20 to 69 into the analysis, resulting in a sample size of *n* = 6228 women (because information on age was only available in 5-year age categories starting with the age group of 15–19 years, we had to exclude women aged 19 years from the analysis).

### 2.2. Variables

In the survey, the utilization of cervical cancer screening was assessed by a categorical variable referring the last time women had received a Pap smear (“≤12 months”, “1 to <2 years”, “2 to <3 years”, “≥3 years”, and “never”). For the present analysis, the outcome of interest was the use of a Pap smear within the last 3 years before the survey (no or yes).

We examined differences in the utilization of cervical cancer screening in non-migrants and the five largest migrant groups residing in Austria, i.e., with a nationality from or born in a Yugoslav successor state, Turkey, Romania, Hungary, and Germany. Different covariates were taken into account. Aside from age (five-year age groups treated as a continuous measure), we considered the partnership status (living with a partner in the same household vs. not living with a partner in the same household), household income (1st, 2nd, 3rd, 4th, and 5th quintiles), and educational level. In the survey, the educational level was assessed according to the International Standard Classification of Education (ISCED) which we collapsed into the three categories “primary and lower secondary education” (ISCED 0–2), “upper/post secondary/non-tertriary education” (ISCED 3/4), and “tertiary education” (ISCED 5–8). In order to account for health differences between the population groups, we considered the self-rated health status (assessed by means of one Likert-scale item with the response categories ranging from “1—very good” to “5—very poor”) and the presence of chronic diseases (yes or no). To consider contextual differences, we took into account the region (federal state) and the degree of urbanization of the place the respondent resides in.

### 2.3. Analysis

Descriptive differences between the population groups were examined by means of analysis of variance for metric variables and chi-square tests for categorical variables. Multivariable logistic regression, reporting adjusted odds ratios (aOR) and their 95% confidence intervals (CI) as effect estimates, was used to adjust the analysis for the aforementioned potential socioeconomic differences as well as differences in health status between the population groups. Intersectional differences were studied by means of interaction terms included into the model and were presented as predicted probabilities [35]. All analyses were conducted using Stata 15 [36].

## 3. Results

A total of 6228 women of the sample were between 20 and 69 years old. Of these, 257 were migrants from a Yugoslav successor state, 158 were German, 103 were Turkish, 70 were Romanian, and 64 were Hungarian migrants. The population groups differed in some of the covariates. On average, migrant women were younger than native Austrian women as evidenced by a lower proportion of individuals in higher age groups. They also more often lived in densely populated areas. Particularly Turkish migrant women had a lower socioeconomic status, with 71.8% having only primary or lower secondary education and 49.5% being below the second quintile in terms of income as compared to 15.1% and 36.9%, respectively, among non-migrant women (Table 1).

Migrant and non-migrant women differed with respect to participation in cervical cancer screening. Whereas 59.6% of non-migrant women had utilized cervical cancer screening in the last three years before the survey, the proportions were lower for Turkish (45.6%), Romanian (51.4%), and Hungarian migrant women (54.7%). Smaller differences were observed in the utilization of cervical cancer screening between non-migrant women, German migrant, and migrant women from a Yugoslav successor state.

Disparities in the utilization of cervical cancer screening between Turkish migrant and non-migrant women also remained after adjustment for the role of socioeconomic and health-related variables, with Turkish migrant women being at 40% lower odds of a utilization in the last three years than non-migrant women (aOR = 0.60; 95% CI: 0.40, 0.91). Although odds were also lower for Romanian (aOR = 0.71; 95% CI: 0.44, 1.16) and Hungarian migrant women (aOR = 0.73; 95% CI: 0.44, 1.20), these differences were smaller and not statistically significant. Aside from the degree of urbanization, a lower utilization of cervical cancer screening was also associated with lower education, higher age, not living with a partner in the same household, and not having any chronic diseases (Table 2).

**Figure 1:**
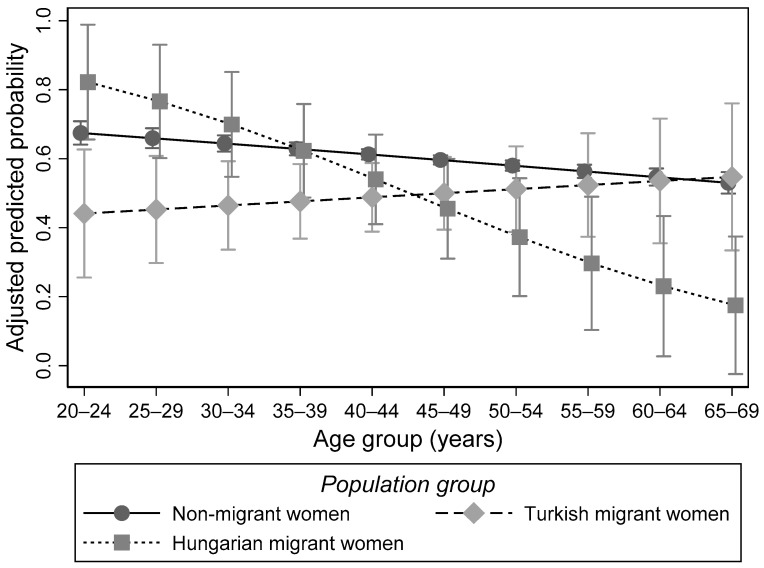
Probability of the utilization of cervical cancer screening by population group and age. Results of the multivariable logistic regression model with the utilization of cervical cancer screening in the last 3 years as the dependent variable and interaction effects between age and population group. Predicated probabilities (Austrian Health Interview Survey 2019; women aged 20–69 years; *n* = 6228).

Intersectional differences could only be observed with respect to age, as becomes evident by the investigation of interaction terms included into the main effects model. Differences in the utilization of cervical cancer screening between Turkish migrant and non-migrant women were particularly pronounced for younger age groups and decreased with age. In contrast, disparities between non-migrant and Hungarian migrant women tended to increase with age (Figure 1).

## 4. Discussion

Studies from several countries have shown that migrants, on average, utilize preventive health care less often than the majority populations of the countries they reside in [37,38,39,40]. This is also the case for cancer screening, including cervical cancer screening [13,14,15,16,17,41,42,43]. One major limitation of many of these studies, however, is that the diversity of the migrant population in terms of cultural background or country of origin could not be taken into account. The present study used a nationwide population-based cross-sectional survey from Austria to examine differences in cervical cancer screening participation between non-migrant women and migrant women from different countries of origin. The results showed that particularly Turkish migrant women utilize cervical cancer screening less frequently than non-migrant women. For Romanian, Hungarian, German, and migrant women from a Yugoslav successor state, disparities are smaller and not statistically significant. This finding is in line with other studies on preventive services, including cancer screening, which illustrate that Turkish migrants are particularly vulnerable in terms of the non-utilization of prevention. For example, a study from the Netherland found out that only 48.0% of women born in Turkey actively participate in cervical cancer screening, whereas 56.8% of native born women actively participate in cervical cancer screening [44]. Other studies from the Netherlands examining differences in the uptake of breast cancer screening between migrant and non-migrant women also demonstrate a lower participation in screening among women of Turkish descent [41,45].

The present study further illustrates that disparities differ with age, with the role of age being different for different groups of migrants. It reveals that for Hungarian migrant women differences in the use of cervical cancer screening compared to non-migrant women increase with age. This has also been indicated by previous research suggesting that older migrants are more disadvantaged than younger ones [46,47]. For Turkish migrant women, however, the present study shows an opposite effect. A significantly lower utilization among migrant women as compared to non-migrant women became only evident for younger age groups. Although this contradicts previous research, similar findings with respect to intersectional differences have been reported in some studies [30,46]. For example, for the utilization of tertiary-preventive health care, differences between Turkish migrants and non-migrants in Germany were reported to decrease with age. Turkish migrants aged 35 years, for instance, were about 15 percentage points less likely than non-migrants to have used rehabilitation before disability-related retirement. At age 50, the difference amounted to only 6 percentage points. For EU migrants, in contrast, the probability of claiming rehabilitation in the five years preceding their disability-related retirement decreased with age relative to non-migrants. No significant interaction effects were observed for other population groups [30].

Non-participation of Turkish migrant women in cervical cancer screening may be attributed to different factors comprising limited proficiency of the language of the country of residence, limited satisfaction with primary health care, and a lack of perceived symptoms [48]. Women with a nationality from or born in a Yugoslav successor state, Romania, or Hungary could have a better command of the local language than women of Turkish origin, which may explain the lower differences as compared to non-migrant women. Studies show that particularly Turkish migrant women often have a low proficiency of German [49]. In addition, limited health literacy regarding cancer screening may be a relevant factor for a reduced participation in corresponding services. In terms of mammography, a study from Germany shows that Turkish migrant women have the lowest level of knowledge about mammography screening as compared to other migrant groups, who have only slightly lower knowledge scores than non-migrant women [50]. Additionally, studies from several countries indicate that a lower awareness of HPV and limited knowledge about HPV vaccination may contribute to lower vaccination rates among migrant women as compared to non-migrant women [51,52,53]. This increases cervical cancer risks for migrant women and further exacerbates risks associated with a poor utilization of cervical cancer screening.

Furthermore, as also the data of the present study reveals, women of Turkish origin, on average, have a lower socioeconomic status, which is associated with a lower participation in cancer screening [7,8,9]. Although we accounted for differences in socioeconomic status, residual confounding cannot be ruled out. Other reasons for lower cervical cancer screening participation may include beliefs and expectation of health care users which are not adequately addressed by health care providers. For example, if cervical cancer screening is performed by male health care staff, this may constitute a significant barrier for some, especially Muslim, women because of perceived shame and embarrassment [10,11]. Providing possibilities to receive services provided by female health care staff is, therefore, an important component of culturally sensitive health care [54].

Given the role of language barriers and knowledge about cervical cancer screening (as well as HPV vaccination), efforts to increase cervical cancer screening participation among Turkish migrant women must therefore also comprise strategies to increase the health literacy of this population group and to provide accessible bilingual information through multi-language materials as well as interpreters [55]. Some health care institutions also provide cross-cultural training for their staff in order to better address their patients’ cultural and religious needs [56]. These approaches aim to better cater to the needs of migrants by providing services which are sensitive to the heterogeneity of expectations, thus reducing the social distance between health care users and health care facilities. As the present study illustrates, these approaches must also take potential intersectional differences into account, which result from the interrelationship of different diversity characteristics. Prior to implementation in routine care, these approaches also need to be thoroughly evaluated with respect to their effectiveness and acceptance by health care users and staff.

The strengths of the present study are the large sample size and the high quality of data collection. Although information is based on self-report, studies conducted on health surveys in Germany which follow a similar approach as the Austrian Health Interview Survey studies that self-reported information corresponds to administrative data and can be considered valid [57]. In addition, the analysis was stratified by different countries of origin, thus accounting for the heterogeneity of the migrant population. A major limitation of the present study is that the Austrian Health Interview Survey was conducted in German language only, thus excluding individuals with very poor German-language proficiency. Given that particularly migrants are affected by limited German-language proficiency and considering its role as a significant barrier in the use of health services [58,59], disparities in the utilization of cervical cancer screening between migrant and non-migrant women demonstrated in the present study may have been underestimated. Furthermore, although we were able to take different countries of origin into account, we could not consider the heterogeneity in terms of culture, religion, acculturation and length of stay, which may also be associated with the utilization of cervical cancer screening [15,16,60].

## 5. Conclusions

To the best of our knowledge, this is the first study in Austria to examine differences in the utilization of cervical cancer screening between non-migrant and different groups of migrant women. It highlights the need to consider the heterogeneity of migrant populations in order to support particularly disadvantaged population groups in making informed decisions and to provide them with appropriate access to preventive care. Reducing existing barriers makes a significant contribution to maintaining and promoting the health and opportunities for migrant communities. The implementation and evaluation of appropriate strategies for this purpose is a central responsibility of health care systems. Given the heterogeneity of migrants, only services sensitive to the diversity of this population group which also take into account intersectional differences allow providing sustainable care that meets the needs of the entire population.

## Figures and Tables

**Table 1:** Description of the study sample by population group (Austrian Health Interview Survey 2019; women aged 20–69 years, *n* = 6228).

	Population Group							*p*-Value *
	Non-Migrant Women	Migrant Women from a Yugoslavian Successor State	German Migrant Women	Turkish Migrant Women	Romanian Migrant Women	Hungarian Migrant Women	Other Migrant Women	
*n*	5199	257	158	103	70	64	377	
*Age (years)*								<0.001
20–24	385 (7.4%)	21 (8.2%)	9 (5.7%)	10 (9.7%)	3 (4.3%)	2 (3.1%)	19 (5.0%)	
25–29	450 (8.7%)	34 (13.2%)	14 (8.9%)	8 (7.8%)	5 (7.1%)	5 (7.8%)	42 (11.1%)	
30–34	416 (8.0%)	30 (11.7%)	20 (12.7%)	11 (10.7%)	10 (14.3%)	12 (18.8%)	51 (13.5%)	
35–39	444 (8.5%)	23 (8.9%)	19 (12.0%)	13 (12.6%)	16 (22.9%)	11 (17.2%)	49 (13.0%)	
40–44	454 (8.7%)	26 (10.1%)	17 (10.8%)	15 (14.6%)	10 (14.3%)	10 (15.6%)	57 (15.1%)	
45–49	539 (10.4%)	30 (11.7%)	18 (11.4%)	14 (13.6%)	8 (11.4%)	10 (15.6%)	46 (12.2%)	
50–54	641 (12.3%)	31 (12.1%)	28 (17.7%)	17 (16.5%)	10 (14.3%)	3 (4.7%)	41 (10.9%)	
55–59	694 (13.3%)	27 (10.5%)	18 (11.4%)	5 (4.9%)	2 (2.9%)	5 (7.8%)	30 (8.0%)	
60–64	639 (12.3%)	18 (7.0%)	11 (7.0%)	3 (2.9%)	6 (8.6%)	1 (1.6%)	26 (6.9%)	
65–69	537 (10.3%)	17 (6.6%)	4 (2.5%)	7 (6.8%)	0 (0.0%)	5 (7.8%)	16 (4.2%)	
*Partnership status*								0.26
Living together with a partner	3502 (67.4%)	177 (68.9%)	115 (72.8%)	78 (75.7%)	52 (74.3%)	42 (65.6%)	264 (70.0%)	
Not living together with a partner	1697 (32.6%)	80 (31.1%)	43 (27.2%)	25 (24.3%)	18 (25.7%)	22 (34.4%)	113 (30.0%)	
*Educational level*								<0.001
Primary/lower secondary	786 (15.1%)	75 (29.2%)	6 (3.8%)	74 (71.8%)	14 (20.0%)	5 (7.8%)	61 (16.2%)	
Upper secondary/post-secondary (non-tertiary)	2879 (55.4%)	131 (51.0%)	93 (58.9%)	24 (23.3%)	36 (51.4%)	30 (46.9%)	142 (37.7%)	
Tertiary education (bachelor, master, and doctoral)	1534 (29.5%)	51 (19.8%)	59 (37.3%)	5 (4.9%)	20 (28.6%)	29 (45.3%)	174 (46.2%)	
*Net income of respondent’s household*								<0.001
Below the 1st quintile	951 (18.3%)	66 (25.7%)	34 (21.5%)	23 (22.3%)	14 (20.0%)	14 (21.9%)	100 (26.5%)	
Between the 1st and 2nd quintiles	966 (18.6%)	46 (17.9%)	20 (12.7%)	28 (27.2%)	12 (17.1%)	13 (20.3%)	74 (19.6%)	
Between the 2nd and 3rd quintile	1301 (25.0%)	70 (27.2%)	36 (22.8%)	28 (27.2%)	31 (44.3%)	21 (32.8%)	99 (26.3%)	
Between the 3rd and 4th quintiles	1105 (21.3%)	56 (21.8%)	35 (22.2%)	19 (18.4%)	8 (11.4%)	13 (20.3%)	67 (17.8%)	
Between the 4th and 5th quintiles	876 (16.8%)	19 (7.4%)	33 (20.9%)	5 (4.9%)	5 (7.1%)	3 (4.7%)	37 (9.8%)	
*Degree of urbanization*								<0.001
High	757 (14.6%)	90 (35.0%)	35 (22.2%)	41 (39.8%)	19 (27.1%)	18 (28.1%)	190 (50.4%)	
Moderate	1687 (32.4%)	107 (41.6%)	57 (36.1%)	49 (47.6%)	26 (37.1%)	23 (35.9%)	109 (28.9%)	
Low	2755 (53.0%)	60 (23.3%)	66 (41.8%)	13 (12.6%)	25 (35.7%)	23 (35.9%)	78 (20.7%)	
*Region (federal state) of residence*								<0.001
Burgenland/Lower Austria	1174 (22.6%)	38 (14.8%)	13 (8.2%)	13 (12.6%)	14 (20.0%)	15 (23.4%)	51 (13.5%)	
Vienna	454 (8.7%)	59 (23.0%)	17 (10.8%)	26 (25.2%)	12 (17.1%)	18 (28.1%)	145 (38.5%)	
Carinthia	314 (6.0%)	12 (4.7%)	10 (6.3%)	1 (1.0%)	5 (7.1%)	5 (7.8%)	14 (3.7%)	
Styria	970 (18.7%)	31 (12.1%)	17 (10.8%)	4 (3.9%)	18 (25.7%)	9 (14.1%)	33 (8.8%)	
Upper Austria	999 (19.2%)	55 (21.4%)	20 (12.7%)	19 (18.4%)	14 (20.0%)	6 (9.4%)	52 (13.8%)	
Salzburg	329 (6.3%)	22 (8.6%)	16 (10.1%)	5 (4.9%)	3 (4.3%)	0 (0.0%)	23 (6.1%)	
Tyrol	646 (12.4%)	23 (8.9%)	39 (24.7%)	18 (17.5%)	1 (1.4%)	8 (12.5%)	35 (9.3%)	
Vorarlberg	313 (6.0%)	17 (6.6%)	26 (16.5%)	17 (16.5%)	3 (4.3%)	3 (4.7%)	24 (6.4%)	
*Self-rated health (1—“very good” to 5—“very poor”) (mean, SD)*	1.8 (0.9)	2.1 (1.0)	1.8 (0.8)	2.3 (1.0)	2.1 (0.8)	1.9 (0.9)	1.9 (0.9)	<0.001
*Presence of chronic disease*								0.065
Yes	1861 (35.8%)	103 (40.1%)	47 (29.7%)	45 (43.7%)	20 (28.6%)	17 (26.6%)	128 (34.0%)	
No	3338 (64.2%)	154 (59.9%)	111 (70.3%)	58 (56.3%)	50 (71.4%)	47 (73.4%)	249 (66.0%)	
*Use of a pap smear test in the last 3 years before the survey*								<0.001
No	2099 (40.4%)	107 (41.6%)	62 (39.2%)	56 (54.4%)	34 (48.6%)	29 (45.3%)	197 (52.3%)	
Yes	3100 (59.6%)	150 (58.4%)	96 (60.8%)	47 (45.6%)	36 (51.4%)	35 (54.7%)	180 (47.7%)	

*Note*. SD: standard deviation. * *p*-value from chi-square test for categorical variables and analysis of variance for continuous variables.

**Table 2:** Results of the multivariable logistic regression model with the utilization of cervical cancer screening in the last 3 years as the dependent variable: adjusted odds ratios (aOR) and 95% confidence intervals (95% CI) (Austrian Health Interview Survey 2019, women aged 20–69 years, *n* = 6228; main effects model. No interaction effects included.).

	aOR	95% CI
*Population group* (Ref.: non-migrant women)		
Migrant women from a Yugoslav successor state	0.95	0.73, 1.24
German migrant women	0.88	0.63, 1.22
Turkish migrant women	0.60	0.40, 0.91
Romanian migrant women	0.71	0.44, 1.16
Hungarian migrant women	0.73	0.44, 1.20
Other migrant women	0.55	0.44, 0.69
*Age*	0.91	0.89, 0.93
*Partnership status* (Ref.: living together with a partner)		
Not living together with a partner	0.74	0.65, 0.84
*Educational level* (Ref.: primary/lower secondary)		
Upper secondary/post-secondary (non-tertiary)	1.35	1.16, 1.58
Tertiary education (bachelor, master, doctoral)	1.52	1.27, 1.82
*Net income of the respondent’s household* (Ref.: below the 1st quintile)		
2nd-income-quintile group	1.01	0.86, 1.20
3rd-income-quintile group	0.95	0.80, 1.13
4th-income-quintile group	1.16	0.96, 1.39
5th-income-quintile group	1.11	0.91, 1.36
*Degree of urbanization* (Ref.: high)		
Moderate	0.97	0.77, 1.22
Low	0.98	0.79, 1.23
*Region (federal state) of residence* (Ref.: Burgenland/Lower Austria)		
Vienna	1.03	0.78, 1.37
Carinthia	1.46	1.13, 1.87
Styria	0.82	0.70, 0.97
Upper Austria	1.00	0.85, 1.18
Salzburg	0.88	0.69, 1.11
Tyrol	1.38	1.14, 1.67
Vorarlberg	1.11	0.88, 1.42
*Self-rated health* (1—“very good” to 5—“very poor”) (mean, SD)	0.93	0.86, 1.00
*Presence of chronic disease* (Ref.: Yes)		
No	0.80	0.70, 0.91

Note: Ref.: reference; SD: standard deviation.

## Data Availability

The data used in the present study can be obtained from Statistics Austria free of charge upon reasonable request. (https://www.sozialministerium.at/Themen/Gesundheit/Gesundheitssystem/Gesundheitsberichte/%C3%96sterreichische-Gesundheitsbefragung-2014-(ATHIS).html (accessed on 31 October 2021); information is only available in German language).
